# Apolipoprotein AI prevents regulatory to follicular helper T cell switching during atherosclerosis

**DOI:** 10.1038/s41467-018-03493-5

**Published:** 2018-03-15

**Authors:** Dalia E. Gaddis, Lindsey E. Padgett, Runpei Wu, Chantel McSkimming, Veronica Romines, Angela M. Taylor, Coleen A. McNamara, Mitchell Kronenberg, Shane Crotty, Michael J. Thomas, Mary G. Sorci-Thomas, Catherine C. Hedrick

**Affiliations:** 1 Division of Inflammation Biology, La Jolla Institute for Allergy and Immunology, 9420 Athena Circle, La Jolla, CA 92037 USA; 2 Cardiovascular Research Center and Division of Cardiology, University of Virginia, 415 Lane Road, Charlottesville, VA 22908 USA; 3 Division of Developmental Immunology, La Jolla Institute for Allergy and Immunology, 9420 Athena Circle, La Jolla, CA 92037 USA; 4 Division of Vaccine Discovery, La Jolla Institute for Allergy and Immunology, 9420 Athena Circle, La Jolla, CA 92037 USA; 5 Division of Infectious Diseases, Department of Medicine, UCSD School of Medicine, 9500 Gilman Drive, La Jolla, CA 92093 USA; 6 Department of Pharmacology and Toxicology, Medical College of Wisconsin, 8701 Watertown Plank Rd, Milwaukee, WI 53226 USA; 7 Department of Medicine, Division of Endocrinology, Medical College of Wisconsin, 9200W. Wisconsin Ave., Milwaukee, WI 53226 USA

## Abstract

Regulatory T (Treg) cells contribute to the anti-inflammatory response during atherogenesis. Here we show that during atherogenesis Treg cells lose Foxp3 expression and their immunosuppressive function, leading to the conversion of a fraction of these cells into T follicular helper (Tfh) cells. We show that Tfh cells are pro-atherogenic and that their depletion reduces atherosclerosis. Mechanistically, the conversion of Treg cells to Tfh cells correlates with reduced expression of IL-2Rα and pSTAT5 levels and increased expression of IL-6Rα. In vitro, incubation of naive T cells with oxLDL prevents their differentiation into Treg cells. Furthermore, injection of lipid-free Apolipoprotein AI (ApoAI) into ApoE^−/−^ mice reduces intracellular cholesterol levels in Treg cells and prevents their conversion into Tfh cells. Together our results suggest that ApoAI, the main protein in high-density lipoprotein particles, modulates the cellular fate of Treg cells and thus influences the immune response during atherosclerosis.

## Abstract (web summary)

Regulatory T (Treg) cells contribute to the anti-inflammatory response during atherogenesis. Here Gaddis et al. show that Apolipoprotein AI prevents the conversion of Treg cells into pro-atherogenic T follicular helper cells, and thus regulates the immune response during atherogenesis.

## Introduction

Regulatory T cells (Treg) play an important role during atherosclerosis development. Depletion of Treg exacerbates atherosclerosis in mouse models, while the transfer of Treg prevents disease progression^1–4^. IL-10 and TGFβ also inhibit atherosclerosis development^5–7^. Treg are a dynamic cell population that are reduced in the aorta of mice fed an atherogenic diet, and can increase when mice are then switched to a regular chow diet^8^. Treg can lose Foxp3 and convert into other CD4 T cell subsets^9–11^, indicating the Treg conversion in inflammatory conditions. A recent study by Butcher et al. has shown that Treg can convert to IFNγ^+^ CD4 T cells in older *ApoE*^*−/−*^ mice^12^. Whether Treg conversion is limited to IFNγ^+^ cells or can extend to other pathogenic T cell subsets during atherogenesis, and understanding the factors that govern this conversion need to be determined.

Apolipoprotein AI (ApoAI) is the major structural protein of plasma HDL. Without ApoAI, plasma HDL concentrations are dramatically reduced^13^. ApoAI is made by hepatocytes and before its release into the plasma interacts on the plasma membrane with ABCA1 to acquire phospholipids and cholesterol to form nascent HDL or pre-HDL particles ABCA1^14–16^. The formation of pre-HDL promotes cholesterol efflux from cells, and thereby stimulates the process of reverse cholesterol transport. Because of ApoAI’s inherent ability to form cholesterol-rich nascent HDL particles, its anti-inflammatory properties have been associated with changes in lipid raft composition, which can modulate immune cell signaling and proliferation^17,18^. The anti-inflammatory role of ApoAI is documented in multiple inflammatory conditions, including lupus^19^, Alzheimer’s disease^20^ and dermatitis^21^. ApoAI can also decrease the maturation of dendritic cells in a way that dampens T cell activation^22^, suggesting that ApoAI can also indirectly influence T cell responses during inflammation.

The relationship between ApoAI and Treg is poorly understood. A study by Wilhelm et al. showed that administration of ApoAI to ApoAI^*−/−*^LDLR^*−/−*^ mice resulted in a decrease in T effector to Treg ratios in the skin draining lymph nodes, and reduced the number of skin-infiltrating T cells in these mice^23^. Can ApoAI influence Treg plasticity during atherogenesis? If yes, what are the mechanisms involved? In this study, we sought to determine the fate of Treg during atherogenesis and how ApoAI affected this process. Collectively, our results show novel findings regarding Treg plasticity and their conversion to T follicular helper cells during atherogenesis and indicate a role for ApoAI in regulating this Treg conversion, shedding light on a collaborative effort between cholesterol metabolism and Treg homeostasis that dampens pro-atherogenic immune responses.

## Results

### ExTreg cells convert to Tfh cells during atherogenesis

**Fig. 1:**
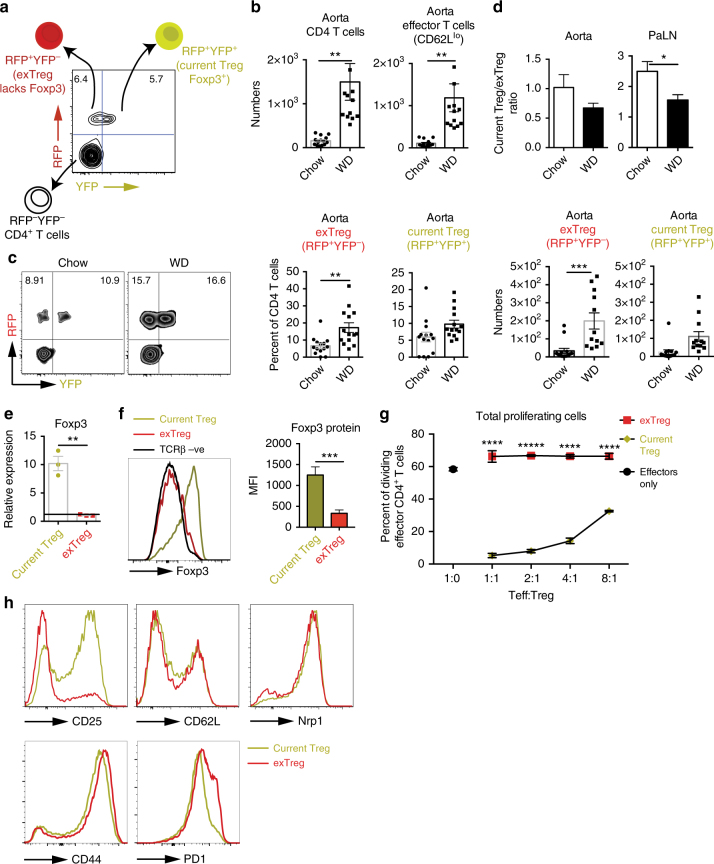
ExTreg cells are increased during atherogenesis. **a** Schematic diagram with a representative flow cytometry plot of the Treg lineage tracker-ApoE^*−/−*^ (LT-ApoE^*−/−*^) mouse model used to track Treg development during atherosclerosis. The diagram shows two population of Treg; current Treg (RFP^+^YFP^+^) cells, which express Foxp3, and exTreg (RFP^+^YFP^−^) cells, which previously expressed Foxp3. **b**–**d** LT-ApoE^*−/−*^ mice were fed a western diet for 15 weeks. Bar graphs compare the numbers of total CD4 T cells and effector CD62L^lo^ cells (**b**), the percentages and numbers of exTreg and current Treg (**c**) in the aorta, and the ratio of current Treg to exTreg in the aorta and PaLN (**d**) of western fed-diet to chow controls. **c** Representative flow cytometry plots and graphs showing the percentages of exTreg and current Treg in the aorta of the above mice. (**e**) Current and exTreg cells were sorted from the PaLN of LT-ApoE^*−/−*^ mice and mRNA levels for *Foxp3* were examined in the extracted RNA and normalized to *β-actin*. **f** Foxp3 protein expression levels in current Treg, exTreg cells and TCRβ negative cells. Representative flow cytometry histograms and bar graphs show the MFI of Foxp3 in the two cell populations. **g** Treg suppression assay using sorted current and exTreg from spleens and peripheral LNs from LT-ApoE^*−/−*^ mice. Sorted current and exTreg cells were cultured with CD4 depleted feeder cells from spleens of B6 mice and increasing numbers of naive effector CD45.1 CD4 T cells (CD25^−^CD44^lo^CD62L^hi^) that were labeled with Cell Trace Violet (CTV). Cells were stimulated with soluble αCD3 for 4 days, harvested and effector cells (CD4^+^CD45.1^+^) were examined for the dilution of CTV by flow cytometry. **h** Flow cytometry histograms showing expression pattern of CD25 (IL-2Rα), CD62L, Nrp1, CD44 and PD1 on current Treg and exTreg cells. Results are expressed as mean ± s.e.m. from two independent experiments (*n* = 14) (**b**–**d**), a representative of two independent experiments (*n* = 3) (**e**, **g**), and a representative of two independent experiments (*n* = 11) (**f**). Statistical significant differences were at *P < 0.05, **P < 0.01, ***P < 0.001, and ****P < 0.0001 (unpaired Student’s *t*-test)

In order to be able to track Treg during atherosclerosis and since Foxp3 is the marker that defines Treg, we needed to create a mouse model that allowed us to track Treg despite Foxp3 expression, on the assumption that Treg may lose Foxp3 expression during atherogenesis. Thus, we developed a novel Treg lineage tracker mouse model; *Foxp3-IRES-YFP-Cre-Rosa26-loxp-td-RFP-loxp-ApoE*^*−/−*^ (LT-ApoE^*−/−*^) and studied these mice in the context of atherosclerosis. In this mouse model, Treg express YFP and Cre recombinase under the control of an IRES element that follows the expression of the *Foxp3* fusion gene. Cre recombinase deletes the *loxp* sites that flank RFP, marking Treg red as well. In this mouse model, “current Treg” cells, which express Foxp3, are both yellow and red. If Treg lose Foxp3 expression, they become an “exTreg”, where they lose YFP expression but retain RFP expression (Fig. 1a). The original Foxp3-IRES-YFP-Cre mice were described in Rubtsov et al.^24^. Using flow cytometry, we can identify and track both current and exTreg cells in the aorta and lymphoid tissues in vivo and can determine the fate of Treg during atherogenesis.

When LT-ApoE^*−/−*^ mice were fed a western diet for 15 weeks, we observed an increase in the number of CD4^+^ T cells in the aorta and most of these cells were activated, CD62L^lo^ cells (Fig. 1b), suggesting that in our mouse model there is CD4 T cell participation during atherosclerosis development. We found an increase (~2 fold) in the percentages of exTreg cells (RFP^+^YFP^−^ CD4 T cells) (Fig. 1c) in the aorta of these mice (See Supplementary Fig. 1 for gating strategy). There were no significant changes in the percentages of current Treg (RFP^+^YFP^+^ CD4 T cells) in the aortas (Fig. 1c). However, this increase in exTreg did result in a significant change in the ratio of current:exTreg cells in PaLN (Fig. 1d), indicating alterations of Treg homeostasis in atherosclerosis. To determine if these exTreg retained any Foxp3 expression or suppressive function, we sorted current and exTreg cells based on YFP and RFP expression and measured both Foxp3 expression and suppressive capacity of the Treg populations. As expected, exTreg cells lacked expression of *Foxp3* mRNA (Fig. 1e), as well as Foxp3 protein levels (Fig. 1f; similar to levels of expression in TCRβ negative cells) compared to current Treg. In addition, exTreg had no suppressive capacity compared to current Treg when cultured with effector CD4 T cells in a standard Treg suppression assay (Fig. 1g). When comparing both current and exTreg cells in western diet-fed, atherosclerotic mice, we found that exTreg had lower expression of CD25 (IL-2Rα), as well as increased PD1 expression. There were no differences in expression of Nrp1, CD62L and CD44 between the two cell populations (Fig. 1h and Supplementary Fig. 2). These results suggested that exTreg and current Treg possess different capacities in their response to IL-2 cytokine and/or their susceptibility to T cell exhaustion.

**Fig. 2:**
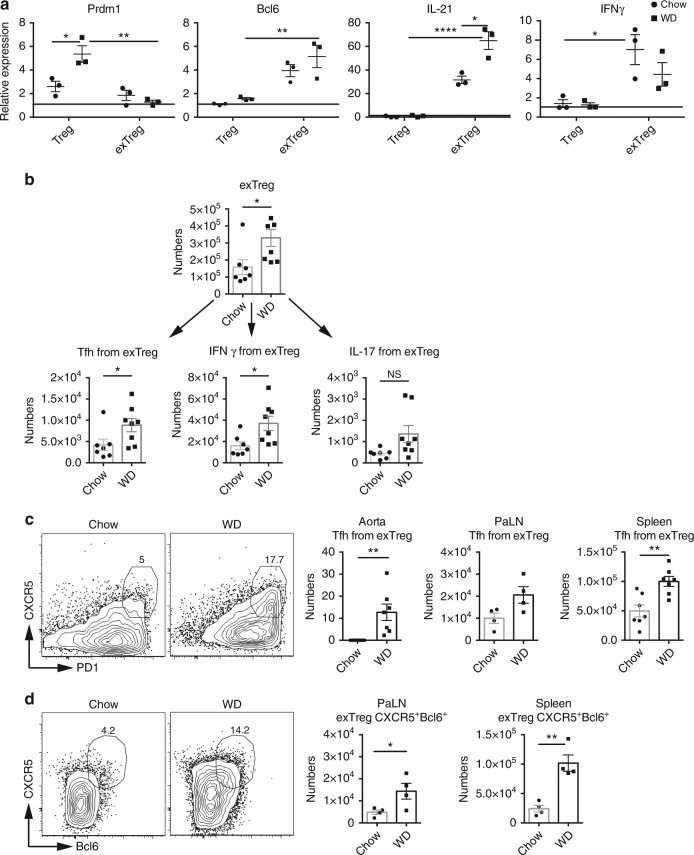
A portion of exTreg population is converting to T follicular helper cells (Tfh) and Th-1 cells. **a** The expression of *Prdm1*, *Bcl6*, *Il21* and *IFNγ* genes by RT-qPCR from RNA extracted from current and exTreg cells sorted from PaLN from chow and western diet-fed LT-ApoE^*−/−*^ mice. The data were analyzed using the 2^−ΔΔCt^ method and it was normalized to *β-actin*. **b** Distribution of different T cell subsets by cell numbers within the exTreg population. Graphs show the number of Tfh, Th1 and Th17 cells within the exTreg population in the PaLN. **c** Representative flow cytometry plots showing the percentages of Tfh (CXCR5^+^PD1^+^) exTreg cells in PaLN of above mice. Graphs show numbers of Tfh exTreg cells in the aorta, PaLN and spleens of above mice. **d** Representative flow cytometry plots showing the percentages of Tfh (CXCR5^+^Bcl6^+^) exTreg cells in PaLN of above mice. Graphs show numbers of Tfh exTreg cells in the PaLN and spleens of above mice. Results are expressed as mean ± s.e.m. from two independent experiments (*n* = 3) (**a**), and mean ± s.e.m from one experiment (n = 7 (chow) and *n* = 8 (WD)) (**b**), and mean ± s.e.m from one experiment (*n* = 7 for aorta and spleen, and *n* = 4 for PaLN) (**c**), and *n* = 4 (**d**). Statistical significant differences were at *P < 0.05, **P < 0.01, and ****P < 0.0001 (two-way ANOVA followed by Tukey’s multiple comparisons test) (**a**) and (unpaired Student’s *t*-test) (**b**–**d**)

Our next goal was to study the exTreg population in more detail to determine if they were phenotypically converting into a different CD4 T cell subset. We investigated the expression of transcription factors related to CD4 T cell function and cytokines present in current and exTreg cells. We found that exTreg cells expressed lower levels of *Prdm1* and higher expression of *Bcl6*, *Il21*, and *IFNγ* compared to current Tregs (Fig. 2a). *Prdm1* encodes Blimp1. Since low Blimp1 expression, accompanied by Bcl6, PD1 (Fig. 1g and Supplementary Fig. 2) and IL-21 expression, are defining features of T follicular helper cells (Tfh)^25^, thus these results indicates that a portion of exTreg cells (RFP^+^YFP^−^) are converting to Tfh and Th-1 cells during atherosclerosis development. We next examined the distribution of Th-1, Tfh and Th-17 cell subsets within the exTreg population in the PaLN of LT-ApoE^*−/−*^ mice fed a western diet. We found that the exTreg population is composed of memory, IFNγ^+^ and Tfh cells (Fig. 2b and Supplementary Fig. 3a) and that the cell numbers of these subsets were increased in atherosclerosis. In addition, these exTreg subsets represented ~12–20% of their respective total cell population showing the relevance of these populations. We tested for Th-17 cells that originated from exTreg cells, but found that less than 0.5% of total exTreg cells produced IL-17 (Fig. 2b and Supplementary Fig. 3a). We also did not observe a significant increase in Th-17 exTreg between chow and western diet-fed mice (Fig. 2b and Supplementary Fig. 3a), suggesting that the role of Th-17 exTreg was negligible in our model.

The role of IFNγ in atherosclerosis has been extensively studied^26–29^ as has Treg:Th-1 plasticity^12^. However, the role of Tfh cells has not been previously demonstrated in atherosclerosis. Therefore, we focused our studies on exploring the causes and consequences of Treg:Tfh plasticity during atherogenesis. As such, we analyzed the exTreg cells (RFP^+^YFP^−^) for activated effector cells that express CXCR5, PD1 and Bcl6 (Tfh are defined as a CXCR5^+^PD1^+^Bcl6^+^CD62L^lo^CD44^hi^ CD4^+^ T cell) (See Supplementary Fig. 1 for gating strategy). In atherosclerosis, we saw increases of 2–4 fold in both percentages and numbers of Tfh cells derived from exTreg cells in the aortas, PaLN and spleens of western diet-fed mice (Fig. 2c, d). Thus, a significant portion of Treg cells converts to Tfh cells during atherosclerosis development.

### Blocking Tfh differentiation reduces exTreg generation

**Fig. 3:**
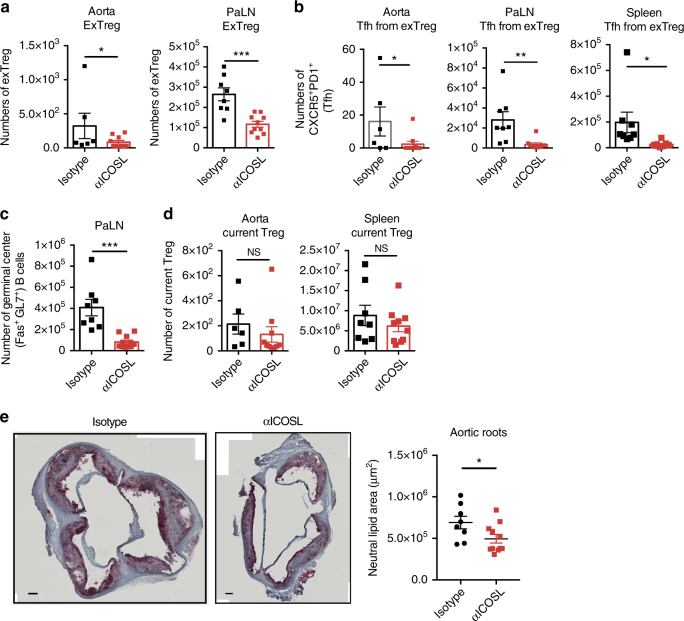
Blocking ICOSL reduces exTreg Tfh cells and atherosclerosis. **a**–**e** LT-ApoE^*−/−*^ mice were fed a western diet for 9 weeks and then were injected with antibody against ICOSL or isotype i.p. (100 μg per mouse, twice per week) for 6 weeks. Graphs show the numbers of exTreg (**a**), exTreg Tfh cells (**b**) in aorta, PaLN and spleen, germinal center B cells in PaLN (**c**) and current Treg cells in aorta and spleen (**d**). **e** Oil Red O stain of aortic roots from the above mice. Images are representative section from each group of mice. Each individual dot in the graph is the average of at least 4 sections per aorta per mouse. Scale bars = 100 μm. Results are expressed as mean ± s.e.m. from two independent experiments (For aorta, *n* = 6 (isotype) and *n* = 10 (αICOSL). For PaLN and spleen, *n* = 8 (isotype) and *n* = 10 (αICOSL). For aortic roots, *n* = 8 (isotype) and *n* = 11 (αICOSL). Statistical significant differences were at *P < 0.05, **P < 0.01, and ***P < 0.001 (unpaired Student’s *t*-test)

Next, we wanted to determine if blocking Tfh differentiation would affect the exTreg population during atherosclerosis progression. ICOS/ICOSL signaling is critical for the differentiation and maintenance of Tfh cells^30^. ICOSL signals to CD4 T cells can be provided by DCs or B cells, and can be blocked with an α-ICOSL mAb^30^. LT-ApoE^*−/−*^ mice were fed a western diet for 15 weeks and given α-ICOSL or isotype antibody twice a week for the last 6 weeks. We found significant reductions in exTreg numbers, of 5 and 2-fold in the aorta and PaLNs, respectively, when mice were treated with α-ICOSL antibody (Fig. 3a). This was accompanied by a 10-fold reduction in the numbers of Tfh cells derived from exTreg in the aorta, PaLNs and spleen (Fig. 3b), as well as a significant decrease in germinal center B cells (Fig. 3c). We observed no differences in current Treg percentages and numbers in αICOSL antibody-treated mice (Fig. 3d). In addition, to their loss of Tfh cells, αICOSL antibody-treated mice showed a 30% reduction in atherosclerosis as measured by Oil Red O staining of aortic roots (Fig. 3e). These results suggested that blocking the interactions that generate Tfh cells is beneficial in reducing the amounts of exTreg Tfh generated during atherogenesis, and suggests that Tfh may be pro-atherogenic.

### Tfh cells are pro-atherogenic

**Fig. 4:**
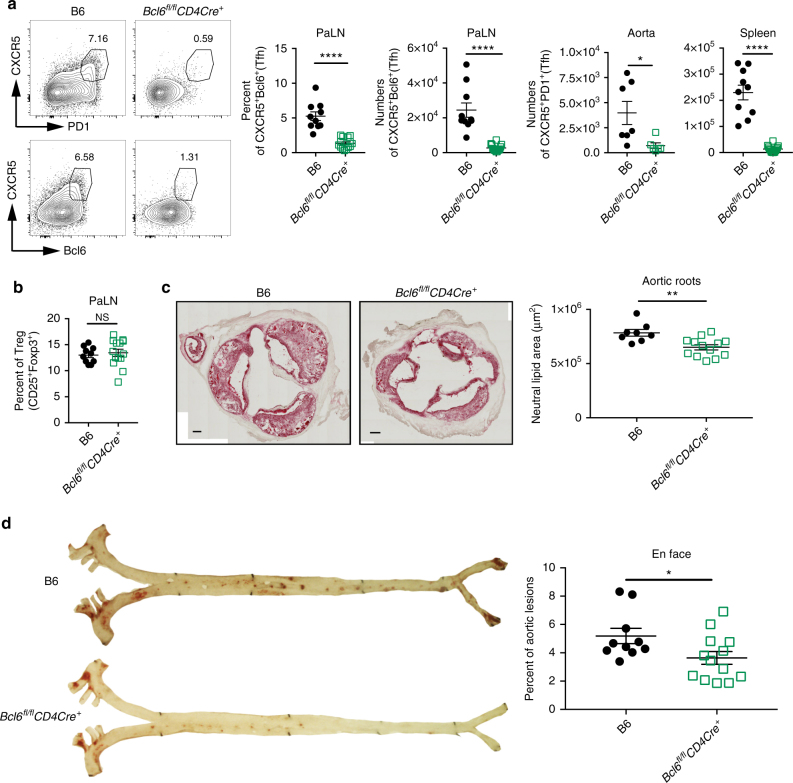
Tfh cells are pro-atherogenic. **a**–**d** B6 or *Bcl6*^*fl/fl*^CD4-Cre^+^ BM chimera mice on *Ldlr*^*−/−*^ background were fed high cholesterol diet for 15 weeks. **a**–**b** Flow cytometry plots and graphs showing the percentages and numbers of Tfh cells in PaLN, aorta and spleen (**a**) and percentages of Treg cells (**b**) in the PaLN. **c** Oil Red O stain of the aortic roots from the above mice. Images are representative sections from each group of mice. The amount of red pixels in each section was determined and the corresponding area was calculated. Each individual dot in the graph is the average of at least 4 sections per aorta per mouse. Scale Bars = 100 µm. **d** En face Oil Red O staining in the aorta of the above mice. Graph shows quantification of the lesion area as a percentage of the total aortic surface area. Results are expressed as mean ± s.e.m. from one experiment (*n* = 12 (B6) and *n* = 14 (*Bcl6*^*fl/fl*^*CD4Cre*^+^) (**a**–**b**), *n* = 8 (B6) and *n* = 13 (*Bcl6*^*fl/fl*^*CD4Cre*^+^) for PaLN and spleen, *n* = 7 (B6) and *n* = 6 (*Bcl6*^*fl/fl*^/*CD4Cre*^+^) for aorta (**c**), and *n* = 10 (B6) and *n* = 13 (*Bcl6*^*fl/fl*^*CD4Cre*^+^) (**d**)). Statistical significant differences were at *P < 0.05, **P < 0.01, and ****P < 0.0001 (unpaired Student’s *t*-test). NS none significant

Because the neutralization of ICOS/ICOSL interactions could impact other cell populations, and to identify whether Tfh cells are indeed directly pro-atherogenic, we further validated our ICOSL blocking experiments using mice that are selectively deficient in Bcl6 in CD4 T cells (*Bcl6*^*fl/fl*^*CD4-Cre*^*+*^)^31^. Bcl6 is the master regulator transcription factor for Tfh cell differentiation, and the lack of this transcription factor in CD4 T cells results in loss of Tfh cells^32–34^. Bone marrow cells from Bcl6^fl/fl^CD4-Cre^+^ and corresponding WT, B6, controls, was used to reconstitute *LdLr*^*−/−*^ mice to generate an atherosclerosis-susceptible animal model that specifically lacked Bcl6 expression in CD4^+^ T cells. Following reconstitution, these mice were fed an atherogenic diet for 15 weeks. The *Bcl6*^*fl/fl*^*CD4-Cre*^*+*^*Ldlr*^*−/−*^ recipients lacked Tfh cells in aorta, PaLN and spleens (Fig. 4a), but had normal Treg percentages compared to control mice (Fig. 4b). *Bcl6*^*fl/fl*^*CD4-Cre*^*+*^*Ldlr* ^*−/−*^ mice exhibited 20% and 30% reductions of atherosclerosis development as determined by Oil red O staining in aortic roots (Fig. 4c) and in entire aortae (Fig. 4d), respectively. Thus, Tfh cells in aorta are directly pro-atherogenic.

To determine if our results were applicable to humans, we measured IL-21, a major Tfh cytokine, in the plasma of human subjects with coronary artery disease (See Supplementary Table 1 for subjects’ characteristics) and the percentage of Foxp3 in Treg (CD4^+^ CD25^+^ CD127^−^). There was a significant inverse correlation between IL-21 plasma levels and Foxp3 expression in Treg cells (Supplementary Fig. 4), validating that similar to mice, in humans Treg and Tfh subsets counterbalanced each other.

### Treg and exTreg Tfh cells differentially express IL-2Rα

**Fig. 5:**
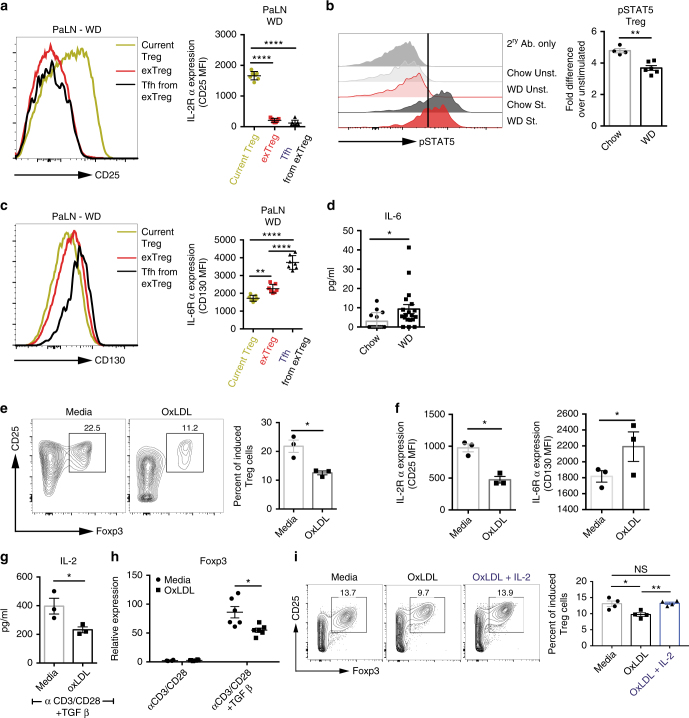
IL-2 and IL-6 receptors expression is altered between current Treg and exTreg Tfh cells. **a** Flow cytometry histograms and graphs of mean fluorescence intensity of IL-2Rα (CD25) expression on current Treg, exTreg and exTreg Tfh cells from LT-ApoE^*−/−*^ mice on western diet. **b** Histogram plots and differential expression of phosphorylated STAT5 by phosphoflow in current Treg cells in the western diet-fed and chow controls LT-ApoE^*−/−*^ following IL-2 stimulation. Graph shows the fold change between unstimulated and IL-2 stimulated samples. **c** Flow cytometry histograms and graph of mean fluorescence intensity of IL-6Rα (CD130) expression on current Treg, exTreg and exTreg Tfh from LT-ApoE^*−/−*^ mice on western diet. **d** Plasma levels of IL-6 in the mice described above. **e**–**i** Naive CD4 T cells from ApoE^*−/−*^ mice were isolated and stimulated with αCD3/CD28 and TGFβ to induce Treg in vitro with or without the addition of oxLDL or IL-2. **e** Flow cytometry plots and graphs showing the percentages of induced Treg following 4 days in culture. **f** Expression of IL-2Rα (CD25) and IL-6Rα (CD130) on total CD4 T cells in culture following 4 days of stimulation. **h**–**g** Supernatant (**h**) and RNA (**g**) were harvested from the above cultures for the assessment of IL-2 by ELISA (**h**) and *Foxp3* mRNA by qRT-PCR (**g**). **i** Flow cytometry plots and graphs showing the percentage of induced Treg with the addition of exogenous IL-2. Results are expressed as the mean ± s.e.m. from one of two independent experiments (*n* = 7) (**a**, **c**), from one of two independent experiments (*n* = 4 (chow) and 6 (WD)) (**b**), from three independent experiments (*n* = 14 (chow) and 18 (WD)) (**d**), from one of two independent experiments (*n* = 3) (**e**–**g**) and (*n* = 6) (**h**), and one of two experiments (*n* = 4) (**i**). Statistical significant differences were at * *P* < 0.05, ** *P* < 0.01, *** *P* < 0.001, and **** *P* < 0.0001 (one-way ANOVA) (**a**, **c**, **i**) and (Unpaired Student’s *t-*test) (**b**, **d**–**h**)

IL-2 plays an important role in the homeostasis of Foxp3 expression and the maintenance of the Treg cells^35^. Constitutively high IL-2Rα expression is a canonical feature of Treg cells. Conversely, IL-2 is an extremely potent inhibitor of Tfh differentiation, mediated by altering expression of Bcl6 and Blimp-1 in a STAT5-dependent manner^36–40^. Our results showed ~ 10-fold reduction in IL-2Rα expression between current and exTreg cells (Fig. 1h and Supplementary Fig. 2); therefore, we examined whether IL-2Rα expression was different between current Treg and Tfh cells in mice fed a western diet. Comparison of IL-2Rα expression on current Treg, exTreg and exTreg Tfh cells in PaLN of western diet-fed mice revealed that IL-2Rα expression was reduced dramatically (~ 20-fold) in Tfh compared to current Treg cells (Fig. 5a). In addition, current Treg from western diet-fed mice displayed reduced ability to phosphorylate STAT5 upon IL-2 stimulation compared to current Treg from chow diet-fed mice (Fig. 5b).

IL-6 plays a key role in early Tfh differentiation through the induction of Bcl6^41–43^. Comparison of IL-6Rα expression on current Treg, exTreg and exTreg Tfh cells showed a stepwise increase in IL-6Rα expression (Fig. 5c). IL-2 and IL-6 can counterregulate expression of IL-6 receptors and IL-2 receptors^44^. Since plasma IL-6 is increased in western diet-fed mice (Fig. 5d), these results suggest that alteration of IL-2 and IL-6 receptor expression in current Treg cells during atherogenesis reduces the ability of Treg cells to receive signals from IL-2, thus, weakening the maintenance of Treg cells, and stimulate Tfh cell generation through the upregulation of IL-6 receptor.

Since low-density lipoprotein (LDL) becomes oxidized in the vessel wall, and contributes to plaque formation during atherogenesis^45,46^, we next isolated naive T cells from *ApoE*^*−/−*^ mice and induced Treg differentiation in vitro with TGFβ with or without addition of oxLDL to determine if oxLDL directly modulated IL-2 and IL-6 receptors on Treg cells. OxLDL reduced iTreg formation (Fig. 5e), which was accompanied by a decrease in IL-2Rα and an increase in IL-6Rα expression (Fig. 5f). In addition, T cells treated with oxLDL produced ~ 30% less IL-2 in culture (Fig. 5g) and had ~ 30% lower *Foxp3* expression (Fig. 5h), observed as early as 12 h following stimulation (Supplementary Fig. 5). Addition of exogenous IL-2 to cultures along with oxLDL increased the percentages of iTreg to levels comparable to without oxLDL (Fig. 5i), thus showing that the reduction in iTreg with oxLDL is due to suboptimal IL-2 levels. These results demonstrate that oxLDL can alter the levels of IL-2 and IL-6 receptor on Treg cells in a manner that impairs Treg homeostasis and favors Tfh cell generation.

### ApoAI reduces exTreg generation during atherogenesis

**Fig. 6:**
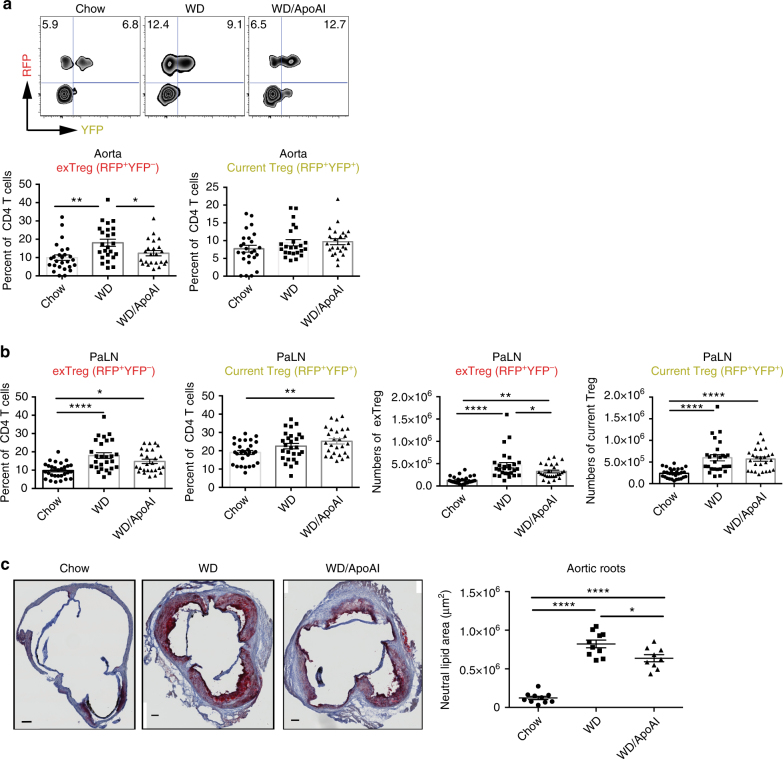
ApoAI administration reduces exTreg during atherogenesis. (**a**, **b**) LT-ApoE^*−/−*^ mice were fed western diet and after 6 weeks, human lipid free ApoAI (500 μg per mouse)(or BSA as control) were given subcutaneously 3 times per week for 9 weeks. Mice were sacrificed after 15 weeks and aorta and PaLN were harvested for analysis. Flow cytometry plots and graphs showing the percentages and/or numbers of RFP^+^YFP^−^ (exTreg) and RFP^+^YFP^+^ (current Treg) in the aorta (**a**) and PaLN (**b**). Results are expressed as the mean ± s.e.m. from four independent experiments (*n* = 29 (Chow), n = 27 (WD) and n = 26 (WD/ApoAI)). **c** Aortic roots from the above mice groups were sectioned and stained with Oil Red O. Images are representative sections from each group of mice. The amount of red pixels in each section was determined and the corresponding area was calculated. Each individual dot in the graph is the average of 8 sections per aorta per mouse. Scale bars = 100 μm. Results are expressed as the mean ± s.e.m. from three independent experiments (*n* = 10 (Chow and WD) and *n* = 9 (WD/ApoAI)). Statistical significant differences were at *P < 0.05, **P < 0.01, and ****P < 0.0001 (one-way ANOVA)

Elevated ApoAI expression in vivo has been associated with reduced Tfh cells^19^ and preservation of Treg cells^23^. We, therefore, tested whether treatment of atherogenic mice with ApoAI could reduce the generation of exTreg and exTreg Tfh cells. LT-ApoE^*−/−*^ mice were fed a western diet for 6 weeks and then given subcutaneous injections of human lipid free ApoAI or BSA (as control) three times per week for an additional 9 weeks. At the end of the 15 weeks, we determined the fate of Treg cells in aorta and PaLN. ApoAI administration reduced the exTreg population in the aorta of western diet-fed mice to almost background chow levels (Fig. 6a). Similarly, in PaLN, ApoAI administration to LT-ApoE^*−/−*^ mice reduced the percentages and numbers of exTreg (Fig. 6b). This was accompanied by an increase in the percentages of current Treg in the PaLN compared to the chow control (Fig. 6b). However, ApoAI had no effect on the total number of Tfh cells (Supplementary Fig. 7b), suggesting that ApoAI prevents the conversion of Treg to exTreg during atherosclerosis progression, rather than affecting the total Tfh differentiation program. ApoAI administration also reduced the atherosclerotic burden in these mice. Mice that were fed a western diet showed increased atherosclerosis, while those treated with ApoAI showed a significant 20% reduction in neutral lipid staining in aortic roots (Fig. 6c). To determine if these findings were relevant in human subjects, we measured ApoAI levels and Treg percentages in blood of human subjects. We found a positive correlation between the levels of ApoAI and the percentages of Treg in blood (Supplementary Fig. 6), further confirming that in mice and in humans, higher levels of ApoAI resulted in an increase in Treg.

### ApoAI prevents the conversion of exTreg to Tfh cells

**Fig. 7:**
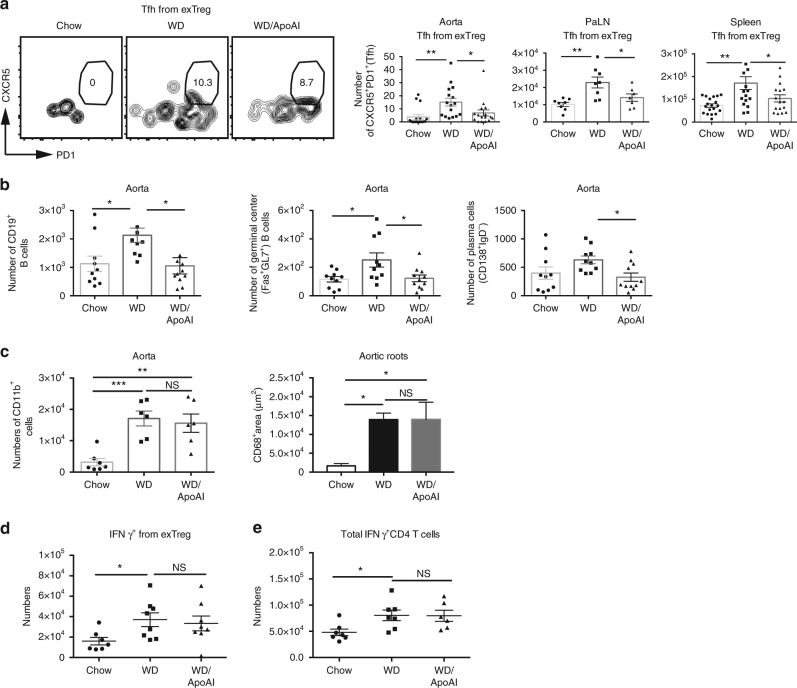
ApoAI selectively prevents the conversion of exTreg into Tfh cells and reduces B cells in the aorta during atherogenesis. **a** Flow cytometry plots and graphs showing percentages and numbers of Tfh cells (CD44^hi^CD62L^lo^CXCR5^+^PD1^+^) from exTreg (CD4^+^RFP^+^YFP^−^) in the aorta, PaLN and spleen in LT-ApoE^*−/−*^ western diet-fed mice with and without ApoAI treatment or chow controls. **b** Graphs showing numbers of B cells (CD19^+^), germinal center B cells (CD19^+^Fas^+^GL7^+^) and plasma cells (CD19^+^CD138^+^IgD^−^) in the aorta of the mice above. **c** Graphs showing numbers of CD11b^+^ cells in the aorta by flow cytometry and area of CD68^+^ in aortic roots by immunofluorescence of the mice above. **d**, **e** Graphs showing numbers of IFNγ^+^ from exTreg, and IFNγ^+^ from total CD4 T cells (**d,** **e**, respectively) in the PaLN of the above mice, following stimulation with PMA/ionomycin for 5 h. Results are expressed as mean ± s.e.m from three independent experiments (*n* = 19 (Chow), *n* = 16 (WD and WD/ApoAI)) (**a**), mean ± s.e.m from two independent experiments (*n* = 10) (b), mean ± s.e.m from one experiment (*n* = 6 (Chow and WD), *n* = 7 (WD/ApoAI)) (**c**) and *n* = 7 (**d**–**e**). Statistical significant differences were at *P < 0.05, **P < 0.01, and ***P < 0.001 (one-way ANOVA)

Since ApoAI reduced exTreg cells generation during atherogenesis, we next determined if ApoAI affected Tfh cell generation from exTreg cells. Administration of ApoAI abolished the increase in exTreg Tfh cells in the aortas of western diet-fed mice to baseline levels found in chow diet-fed mice (Fig. 7a). We saw similar results in the PaLN and the spleens of LT-ApoE^*−/−*^ mice (Fig. 7a). Upon examining B cell populations in the aorta, ApoAI administration reduced the total number of CD19^+^ B cells, germinal center B cells (CD19^+^Fas^+^GL7^+^) and plasma cells (CD19^+^CD138^+^IgD^−^) to levels similar to baseline chow levels (Fig. 7b) (See Supplementary Fig. 8 for gating strategy for B cells in aorta). To exclude the possibility that the observed changes in cell numbers were not merely due to the anti-inflammatory properties of ApoAI, we examined macrophages in the aorta of LT-ApoE^*−/−*^ mice that received ApoAI and we found no reduction in the total numbers of CD11b^+^ cells in the aorta by flow cytometry or by immunofluorescence staining for CD68^+^ cells (Fig. 7c). We also did not observe a reduction in IFNγ^+^ exTreg (Fig. 7d) or total IFNγ^+^ CD4 T cells (Fig. 7e) upon ApoAI administration. In addition, there was no change in total IL-17^+^ CD4 T cells with ApoAI administration (Supplementary Fig. 9). These results suggested that ApoAI was specifically affecting the exTreg to Tfh cell conversion and not other cell types that may be contributing to atherosclerosis development.

### ApoAI increases IL-2Rα on Treg cells

**Fig. 8:**
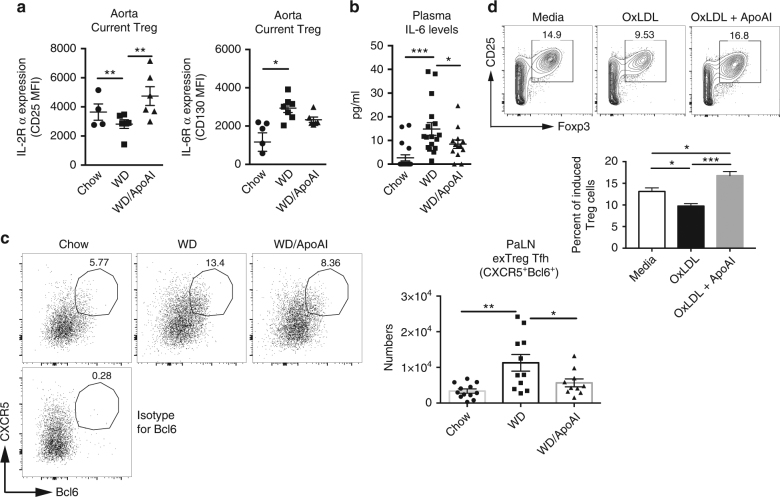
ApoAI administration increases IL-2Rα expression and reduces IL-6 production and Bcl6 expression during atherogenesis. **a** Expression of IL-2Rα (CD25) and IL-6Rα (CD130) on current Treg in the aorta of LT-ApoE^*−/−*^ western diet-fed mice with and without ApoAI treatment or chow controls. **b** Plasma levels of IL-6 in the mice described above. **c** Flow cytometry plots and graphs showing the percentages and numbers of CXCR5^+^Bcl6^+^ Tfh in exTreg cells of above mice. Plots are gated on CD44^+^RFP^+^YFP^−^ CD4 T cells. **d** Naive CD4 T cells from ApoE^*−/−*^ mice were isolated and stimulated with αCD3/CD28 and TGFβ to induce Treg in vitro with or without the addition of oxLDL or ApoAI. Flow cytometry plots and graphs showing the percentages of induced Treg following 4 days in culture. Results are expressed as the mean ± s.e.m. from one of two independent experiments (*n* = 6 (Chow and WD/ApoAI), *n* = 7 (WD)) (**a**), mean ± s.e.m. from 3 different experiments (*n* = 18 (Chow), *n* = 17 (WD) and *n* = 13 (WD/ApoAI)) (**b**), the mean ± s.e.m. from two independent experiments (*n* = 11 (Chow and WD) and *n* = 10 (WD/ApoAI) (**c**), and the mean ± s.e.m. from one of two experiments (*n* = 4) (**d**). Statistical significant differences were at *P < 0.05, **P < 0.01 and ***P < 0.001 (one-way ANOVA)

We next determined if ApoAI administration could affect IL-2Rα and IL-6Rα expression by current Treg cells. ApoAI infusion reversed the decrease in IL-2Rα expression on current Treg cells in western diet-fed mice (Fig. 8a), indicating that ApoAI can reverse the defect in IL-2 signaling induced by western diet. This was accompanied by a decrease in the expression of IL-6Rα on current Treg cells (Fig. 8a) and a reduction in plasma IL-6 levels (Fig. 8b). Administration of ApoAI also reduced the expression of Bcl6 in exTreg Tfh cells (Fig. 8c). Furthermore, addition of ApoAI together with oxLDL during in vitro Treg induction reverses the oxLDL impairment of Treg differentiation (Fig. 8d). Our results suggest that ApoAI reduces the exTreg to Tfh generation through maintenance of IL-2R expression on Treg cells and reduction of IL-6, resulting in reduced Bcl6 expression, correcting the imbalance in these cytokine receptors and transcription factors that drive the Treg:exTreg Tfh conversion during western diet feeding.

### ApoAI reduces cellular cholesterol levels in Treg

**Fig. 9:**
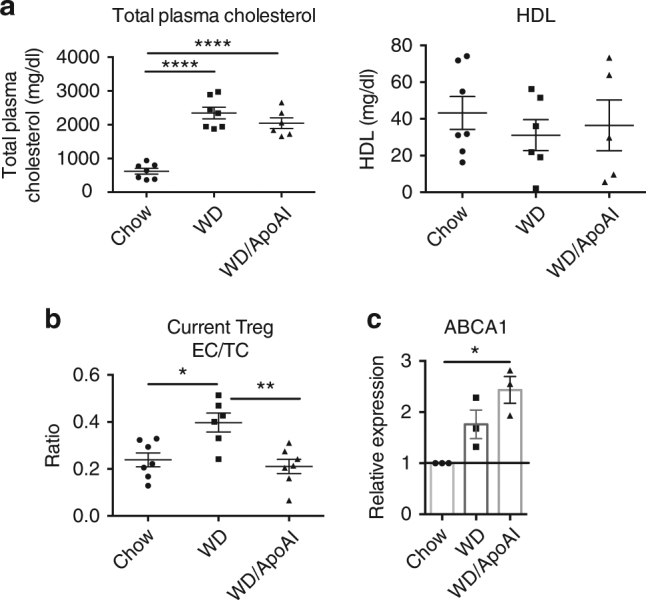
ApoAI administration reduces the cholesterol content in current Treg cells through increasing ABCA1 expression. **a** Plasma from LT-ApoE^*−/−*^ western diet-fed mice with and without ApoAI treatment or chow controls were tested for total cholesterol and HDL. **b** Sorted current Treg (RFP^+^YFP^+^) cells from PaLN of the above mice were analyzed for the amounts of intracellular free and total cholesterol using mass spectroscopy. The amount of esterified cholesterol was calculated followed by the ratio of esterified to total cholesterol. **c** The mRNA expression of *ABCA1* by RT-qPCR from RNA extracted from current Treg sorted from the PaLN of chow and western diet-fed LT-ApoE^*−/−*^ mice, with and without ApoAI treatment. The data were analyzed using the 2^−ΔΔCt^ method and it was normalized to *β-actin*. Results are expressed as the mean ± s.e.m from one experiment (*n* = 7) (**a**, **b**), and mean ± s.e.m from one of two independent experiments (*n* = 3) (**c**). Statistical significant differences were at *P < 0.05, **P < 0.01 and ****P < 0.0001 (one-way ANOVA)

Cholesterol is an important component in membrane lipid rafts where IL-2 receptor is enriched^47^. Thus, we wanted to determine if the changes in IL-2R expression with western diet and ApoAI administration were due to changes in cholesterol levels in current Treg. First, we measured the levels of plasma cholesterol and HDL in our model. No differences in total plasma cholesterol or HDL were observed between the BSA and ApoAI-treated western diet-fed mice (Fig. 9a), showing that it is not merely a function of ApoAI changing the plasma cholesterol or HDL levels. This is consistent with prior studies showing that ApoAI infusion results in transient or low changes in plasma HDL concentration^21,48^. Upon measuring the intracellular cholesterol levels of current Treg, we found an increase in the ratio of esterified cholesterol to total cholesterol (EC/TC) in current Treg from western diet fed mice (Fig. 9b), which was reduced by treatment of mice with ApoAI. These results suggested that with western diet feeding, there was a disruption in cellular cholesterol homeostasis, resulting in the accumulation of esterified cholesterol in Treg. The intracellular cholesterol of Treg was reduced with ApoAI administration (Fig. 9b). Since ApoAI couples with the cholesterol transporter ABCA1 to efflux cellular cholesterol and it has been shown that ApoAI can regulate ABCA1 expression^49^, we next checked for levels of ABCA1 expression. Treatment with ApoAI significantly increased the levels of *ABCA1* mRNA above baseline levels (Fig. 9c). This finding suggests that ApoAI may alter intracellular cholesterol levels by increasing the levels of ABCA1 and thus, increasing efflux through ABCA1 in Treg. Thus, it is likely that the changes in IL-2R and the stability of the Treg population could be at least partly due to changes in intracellular cholesterol levels, which are reversed by the administration of ApoAI due to its ability to facilitate cholesterol efflux cholesterol from cells.

## Discussion

The main objectives of this study were two-fold: (i) to determine the fate of Treg cells during atherosclerosis development, and (ii) to determine how ApoAI affects Treg cell plasticity during atherogenesis. Here, we show two novel findings. First, during atherosclerosis development, a portion of Treg cells switch phenotype into pro-atherogenic Tfh cells. Second, ApoAI prevents Treg to Tfh cell conversion during atherosclerosis.

Our results show that in atherosclerosis, a portion of Treg cells converts to Tfh cells, which are responsible for increasing tertiary lymphoid structures in the aorta, promoting B cell-mediated antibody production^50–52^. The concept of Treg plasticity has been well-documented (reviewed in refs.^53,54^). T cell differentiation depends largely on the extent of antigenic stimulation, the cytokine milieu, and fluctuations in the metabolic and nutrient levels to which the cells are exposed. Treg conversion into inflammatory and pathogenic Th1 or Th17 has been reported in autoimmune diseases and lethal infections (reviewed in^55^). In mouse models of type I diabetes, as well as in type I diabetic patients, exFoxp3 or Foxp3 expressing cells produce IFNγ ^9,11^. Isolated Treg can convert to Tfh cells in Peyer’s patches of mice^56^. A recent report has shown that in old *ApoE*^*−/−*^ mice, IFNγ^+^ Treg exist in the aorta and lack suppressive functions^12^. To our knowledge, this is the first report to show the conversion of Treg into Tfh cells during atherosclerosis. Tfh cells play critical roles in germinal center formation and B cell activation, both of which are important aspects of atherosclerotic disease pathogenesis^51,52^. It is understood how naive T cells become Tfh cells^25^, however, whether Treg: Tfh cells plasticity occurs is controversial^57–59^. Here, we show that increasing intracellular cholesterol levels, either by western diet feeding or oxLDL uptake, reduces the expression and signaling of IL-2R, a potent stabilizer of Treg^35^. IL-2 and pSTAT5 are also potent inhibitors of Tfh differentiation^36,37^, suggesting that cholesterol-mediated IL-2 signaling attenuation, together with increased IL-6R and Bcl6 expression, initiate Tfh differentiation in context of atherosclerosis. When sufficient concentrations of ApoAI are available, ApoAI likely prevents this conversion process by regulating cholesterol levels in Treg cells and sustaining IL-2 receptor expression. Other studies have alluded to the pro-atherogenic role of Tfh cells in atherogenesis. Clement et al. have shown regulatory CD8 T cells can reduce Tfh-B cell activation and limit atherogenesis^51^. More recently, a study by Ziad Mallat’s group elegantly showed that marginal zone B cells limited Tfh cells and decreased atherosclerosis development^60^. In our experiments, we first show that the lack of exTreg Tfh cells via anti-ICOSL blockade results in reduction of atherosclerosis. ICOS signaling is preferentially required for Tfh cell differentiation but does impact additional cell types. Thus, to prove a direct role of Tfh cells in atherogenesis, we used mice that possessed CD4 T cells that were conditionally-deficient in *Bcl6*, the lineage transcription factor of Tfh cells. In these mice, we also saw reduced atherosclerosis burden. Thus, we show definitively using two independent methods in vivo that Tfh cells are pro-atherogenic and the regulation of this population can control atherosclerosis progression.

The protective anti-inflammatory role of lipid-free ApoAI, phospholipid-bound ApoAI (i.e., recombinant HDL) and ApoAI mimetics has been previously documented in atherosclerosis and other inflammatory conditions^17,19,20,23,61^. Recombinant HDL doses used in humans reflect the current dogma that plasma HDL concentrations must increase in order to show efficacy in reducing arterial cholesterol burden. However, animal model studies have shown significant peripheral cholesterol transport following subcutaneous injections of low dose lipid-free human ApoAI that either results in minor or transient increases in plasma HDL concentrations^21,48^. Previously, we have shown that ApoAI administration reduced cholesterol-mediated T cell expansion and increased Treg to effector CD4 T cell ratios in skin draining LNs in *Ldlr*^*−/−*^*ApoAI*^*−/−*^ mice, reversing the autoimmune phenotype seen in these mice^23^. In this current study, we observe no changes in plasma HDL concentration in western diet-fed ApoAI treated mice compared to BSA controls, yet we observe a significant impact of ApoAI on Treg homeostasis. ApoAI changed the intracellular cholesterol levels as monitored by the esterified cholesterol to total cholesterol ratio. Macrophages transduced with human ApoAI led to a reduction in atherosclerosis and dermatitis without affecting plasma HDL levels^21^, further proving that by changing the cellular cholesterol content, ApoAI affects the cellular inflammatory state and, hence, the outcome of inflammatory diseases. Here, we also provide evidence that ApoAI affects Treg plasticity and conversion to Tfh cells. Our results are in agreement with a study showing that ApoAI transgenic mice have reduced Tfh cells and germinal center B cell generation in a Lupus model^19^. Since ApoAI reduces dendritic cell differentiation and maturation^22^, we hypothesize that ApoAI influences Treg maintenance both directly through modulation of cholesterol content as we show here, and indirectly, through dendritic cell inhibition. That said, the athero-protective properties of ApoAI can not be directly linked to the reduction of Tfh cells as other anti-inflammatory properties of ApoAI may play a role. Further studies are needed to tease out if the ApoAI effect on Tfh cells can directly impact atherosclerosis.

One question that arises from our study is whether ApoAI administration is sufficient to rescue T cells from the massive loads of cholesterol in our system. While cells, like endothelial cells have mechanism(s) that regulate their cholesterol intake and they never become foam cells (i.e., cholesterol-ester loaded), other cells are unable to effectively regulate the amount of cholesterol due to extensive scavenger receptors and readily become foam cells (reviewed in ref.^62^). Regardless, the amount of cholesterol per cell needed to elicit dramatic changes in cellular signaling and activation mediated by elevated microdomain cholesterol composition is relatively small. This was recently demonstrated by Kaul et al.,^17^. In addition, the efficacy of subcutaneous injections of lipid-free ApoAI has been previously shown in other reports^23,63^. Human and mouse ApoAI have different primary structures but they both interact with ABCA1 to yield nascent HDL particles which eventually become spherical mature plasma HDL. Mouse plasma HDL is homogenously sized while human plasma HDL tends to be more heterogeneous sized and the structure is likely the basis for this difference^64^. Differences in ApoAI structure relative to cholesterol efflux and nascent HDL formation may explain small differences in efflux, but to focus on bulk plasma HDL size and composition is not the mechanism by which lipid-free ApoAI suppresses atherosclerosis or alters T cell plasticity. Rather, lipid-free ApoAI stimulates ABCA1 removal of excess cellular cholesterol via the formation of nascent HDL particles, thereby suppressing signaling that would otherwise lead to activation and/or recruitment of immune cells to the atherosclerosis plaque. We have previously published that 10% of the subcutaneously injected ApoAI enters plasma in lipidated form^23^. We believe that mouse ApoAI would work equally well as human ApoAI as they both work well in vitro to stimulate efflux. We do not know if both forms of ApoAI are found in plaque. Since atherosclerotic plaques in humans have been shown to harbor large amounts of serum albumin and other abundant plasma proteins that diffuse into the oxidative environment of the plaque where they become modified and degraded. We would assume both human and mouse ApoAI would be found in nearly equal amounts in plaque respectively, although plasma HDL cholesterol concentrations in mice is higher (~65  mg dl^-1^) than in humans (~45  mg dl^-1^) and diffusion into plaques appear to be driven through mass action.

A recent study from our laboratory showed that ABCG1, another cholesterol transporter, regulated Treg development through modulation of cholesterol levels and mTOR activation^65^. The results from both studies suggest that Treg require an optimal level of plasma membrane cholesterol for their development and/or maintenance, and that slight changes in cholesterol levels, affect Treg homeostasis. In this study, our results indicate that cholesterol levels can play a role regulating IL-2 signaling, a critical factor for the maintenance of Treg. Minor changes in plasma membrane lipids can either enhance or abolish IL-2 signaling events^47,66^. Could accumulation of too much cholesterol in Treg be detrimental for their proper function and maintenance? The increase in exTreg and their conversion to Tfh cells in LT-ApoE^*−/−*^ mice fed a western diet, as well as our in vitro Treg induction in presence of oxLDL, would provide evidence that this is true. We hypothesize that cholesterol accumulation in Treg from western diet-fed mice increases lipid micro-domains, decreasing Treg maintenance and allowing for their conversion to Tfh cells, a process that is reversed by ApoAI, in part mediated by its role in increasing reverse cholesterol transport.

Our results show a negative correlation between plasma levels of IL-21 and Foxp3 and a positive correlation between plasma ApoAI and Treg and in human samples obtained from patients with coronary artery disease, indicating that our findings in atherosclerotic mouse models have relevance in humans. Tfh cells and tertiary lymphoid structures have been found in human atherosclerotic aneurysmal arteries^51^. IL-21 and IL-21R positively correlate with inflammatory factors in human adipose tissue, suggesting a potential role for IL-21 in fat tissue^67^. Treg cells are found to be defective in patients with coronary artery disease^68^. Our studies suggest that increasing ApoAI levels in humans could potentially reduce the generation of Tfh cells and increase Treg maintenance in such a way that helps reduce inflammation and the overall outcome of plaque deposition and atherosclerosis. Here, we provide mechanistic insights into how ApoAI may be protective by regulating Treg:Tfh conversion during disease progression. What is not clear is whether increasing HDL concentrations by pathways that do not directly involve ABCA1 (i.e., decreased catabolism, aqueous diffusion of cellular cholesterol to HDL), would be as efficacious as increasing lipid-free or even lipid-poor ApoA-I levels.

Overall, this study uncovers how Treg cells convert into Tfh cells under atherogenic conditions and how ApoAI influences Treg plasticity by altering cholesterol content in these cells. We believe our study opens a new avenue for the future use of lipid-free ApoAI to modulate Treg:Tfh responses that can benefit and be applicable to atherosclerosis, as well as to other inflammatory diseases.

## Methods

### Mice

The original breeding pairs of B6, CD45.1, *ApoE*^*−/−*^, and *LDLR*^*−/−*^ mice were purchased from The Jackson Laboratories (Bar Harbor, Maine; Stock numbers 0000664, 002014, 002052, and 002207, respectively). The original Foxp3-IRES-YFP-CRE-Rosa26-dt-RFP (Treg lineage tracker) was a kind gift from Mitchell Kronenberg (La Jolla Institute for Allergy and Immunology, La Jolla, CA) (LJI). *Bcl6*^*fl/fl*^ mice^69^ crossed to *CD4-Cre*^*+*^ mice were bred at LJI^31^. Treg Lineage tracker mice were crossed in our laboratory to ApoE^*−/−*^ mice to create the LT-ApoE^*−/−*^ mice. Mice were used for experiments at 6-12 weeks of age. Mice of both sexes were used and all mice were randomly selected for different treatment groups. For most experiments, investigators were blinded from the group allocation when analyzing data. For atherosclerosis studies, LT-ApoE^*−/−*^ mice were fed a Western Diet (42% from fat, 0.2% from cholesterol) (Harlan, #TD 88137, Placentia, CA). LDLR^*−/−*^ mice were fed a high-cholesterol diet (1.25% cholesterol and 40% calories from fat) (Research Diets, D12108C). All other mice including chow controls were fed a standard chow diet containing 0% cholesterol and 5% calories from fat (Pico lab, #5053, Saint Louis, MO). All mice were bred and housed in microisolator cages in a pathogen free animal facility of LJI. All experiments followed guidelines of the LJI Animal Care and Use Committee and the approval of the use of rodents was obtained from LJI according to criteria outlined in the Guide for the Care and Use of Laboratory Animals from the National Institutes of Health.

### Flow cytometry

Peri-aortic LNs (PaLN) or spleens from the mice were passed through a 40 μm cell strainers and washed with ice cold PBS. For spleens, RBCs were lysed with 1 × RBCs lysis buffer (Biolegend, San Diego, CA). Aortae were explanted after perfusion with ice cold PBS, cut and were digested with DNAse I, Collagenase type I, Collagenase type XI and Hyaluronidase type I as previously described^65^, then they were passed through 40 μm filters. Cells were incubated with RPMI media with 10% FCS to restore expression of surface markers for at least 30 min at 37 °C. All staining protocols were done on ice for 30 min unless otherwise indicated. Samples were stained with Live Dead fixable dye (Life Technologies, Carlsbad, CA). Samples were acquired using LSRII (BD, Bioscience, San Diego, CA) and data were analyzed using Flowjo Ver 9.8 and 10.0.08 (Tree Star, Ashland, OR).

For surface staining, LT-ApoE^*−/−*^ lymphocytes were stained with antibodies against CD4 (clone RM4-4, 0.06 μg per test, ebioscience), TCRβ (clone H57-597, 1 μg per test, ebioscience), CD25 (clone PC61, 0.06 μg per test, ebioscience) and/or CD62L (clone MEL-14, 0.125 μg per test, ebioscience), CD130 (IL-6Rα; clone KGP130, 0.125 μg per test, ebioscience), Nrp1 (clone N43-7, 0.1 μg per test, MBL international), and CD45 (clone 30-F11; for Aorta, 0.125 μg per test, ebioscience), in cold FACS buffer (2% BSA, 0.01% sodium azide in PBS). YFP and RFP were detected without further staining unless otherwise indicated. For suppression assay, cells were stained with antibodies against CD4, TCRβ, CD25 and CD45.1 (clone A20; for suppression assay, 0.5 μg per test, ebioscience). For Tfh detection, cells were stained initially with CXCR5-Biotin (clone 2G8, 0.5 μg per test, BD bioscience), followed by fluorescent-labeled Streptavidin together with the previous antibodies and CD44 (clone IM7, 0.125 μg per test, Biolegend), CD62L, PD1 (clone J43, 0.25 μg per test, ebioscience) and CD19 (clone 6D5, 0.5 μg per test, Biolegend). For B cells, cells were stained with antibodies against CD19, Fas (clone 15A7, 0.5 μg per test, ebioscience), IgD (clone 11-26c, 0.25 μg per test, Biolegend), GL7 (clone GL-7, 0.5 μg per test, ebioscience), CD138 (clone 281-2, 0.5 μg per test, Biolegend) and CD4, CD8 (clone 53-6.7, 0.25 μg per test, ebioscience), CD11c (clone N418, 0.6 μg per test, ebioscience), Gr-1 (clone RB6-8C5, 0.03 μg per test, ebioscience) and F4/80 (clone BM8, 0.125 μg per test, ebioscience) (for dump gate). For macrophages, CD11b (clone M1/70, 0.5 μg per test, ebioscience) was used.

For Bcl6 Expression, cells were stained for Tfh as above. Cells were fixed and permeabilized and stained with antibodies against Bcl6 (clone K112-91, 5 μl per test, BD Bioscience).

For Treg staining, cells were surface stained with antibodies against CD4, TCRβ and CD25. Cells were fixed and permeabilized with Foxp3 staining buffer kit (Ebioscience). Cells were then stained with antibody against Foxp3 (clone FJK-16S, 0.125 μg per test, ebioscience) in permeabilization buffer.

For intracellular cytokine staining, cells from PaLN were stimulated in RPMI-10% FCS media with PMA and Ionomycin (50 ng ml^−1^ and 1 μg ml^−1^, respectively) (Sigma–Aldrich) for 5 h in the presence of GolgiPlug (BD Biosciences) at 1 μl ml^−1^. Cells were then stained for CD4, TCRβ, CD44, CD19, PD1, and CXCR5 as described above. Cells were fixed for 10 min with 2% freshly prepared paraformaldehyde (Sigma-Aldrich) and then permeabilized as described above. Cells were then stained with antibodies against IFNγ (clone XMG-1.2, 0.05 μg per test, BD bioscience), IL-17 (clone ebio1787, 0.125 μg per test, ebioscience), Foxp3, and rabbit-RFP (polyclonal, 0.25 μg per test, Rockland, Limerick, PA) followed by a stain for donkey anti-rabbit-PE (0.125 μg per test, Biolegend) for the detection of RFP.

For pSTAT5, spleen cells were stimulated with recombinant human IL-2 (100 U ml^−1^) (Peprotech, Rocky Hill, NJ) for 45 min at 37 °C. Cells were surface stained as previously described. Cells were washed and fixed with 1 × Lyse/Fix buffer (BD Biosciences) for 10 min at 37 °C. Cells were washed and chilled on ice and permeabilized with pre-chilled BD phosphoflow Perm Buffer III for 30 min (BD Biosciences). Cells were washed and stained for Foxp3 and rabbit pSTAT5 (D47E7, 1:200, Cell Signaling Technologies, Beverly, MA) followed by anti-rabbit-AF647 (0.125 μg per test, Biolegend).

### Suppression assay

Current and exTreg cells were sorted based on expression of YFP and RFP. Naive effector cells (CD4^+^CD25^−^CD44^lo^CD62L^hi^) cells were sorted from spleens and peripheral LNs of CD45.1 mice and were labeled with cell trace violet (CTV) (Life Technologies) according to manufacturer’s instruction. Cell sorting was done using FACSAria (BD Biosciences). Feeder cells were prepared from spleens of B6 mice and CD4 T cells were depleted using CD4^+^ magnetic beads (Miltenyi Biotec, Auburn, CA) and then were irradiated with 3000 rads. Feeders were cultured with naive effector T cells at a ratio of 3:1 in U-shaped 96 well plates. Sorted current or exTreg cells were added at a ratio of 1:1, 1:2, 1:4, 1:8 or not added for effectors only wells. All wells, except for unstimulated wells, were stimulated with soluble αCD3 antibody at 2.5 μg ml^−1^. All cells were cultured in RPMI media supplemented with 10% FCS, L-glutamine, penicillin and streptomycin, β-mercaptoethanol. Cells were harvested 4 days following stimulation and the percentage of effector CD45.1 cells that diluted CTV was determined.

### In vitro Treg induction

Naive CD4 T cells were sorted from spleens and lymph nodes of ApoE^*−/−*^ as described earlier or using Easy Sep^TM^ Naive T cell isolation kit (STEMCELL Technologies, Inc., Vancouver, Canada). Equal numbers of cells in serum free CST OpTmizer™ T cell expansion media (Life Technologies) supplemented with L-glutamine, penicillin and streptomycin and β-mercaptoethanol were incubated in αCD3 coated plates at 2 μg ml^−1^ concentration. Soluble αCD28 (1 μg ml^−1^) and TGFβ (2 ng ml^−1^) were added to the wells. For oxLDL conditions, 10 μg ml^−1^ of human oxLDL (KalenBiomed, Montgomery Village, MD) was added to the culture. For IL-2 conditions, cells were stimulated with recombinant human IL-2 (100 U ml^−1^). For in vitro ApoAI conditions, 200 μg ml^−1^ final concentration was used. Cells were incubated for 36 h (IL-2 detection or *Foxp3* mRNA) and for 4 days (for induced Treg staining). Staining for Treg was done as indicated earlier.

### Purification of Human plasma ApoAI and administration in LT-ApoE^*−/−*^ mice

Human ApoAI was purified as previously described^23^. ApoAI concentration was adjusted to 2.5–5 mg ml^−1^ with saline. BSA (Sigma, St. Louis, MO) was prepared at the same concentration and filter sterilized to serve as control. After feeding LT-ApoE^*−/−*^ mice a western diet for 6 weeks, mice were injected subcutaneously with 500 μg of ApoAI or BSA. Chow mice also received BSA as controls.

### In vivo ICOSL blocking

LT-ApoE^*−/−*^ mice were fed a western diet for 15 weeks. For the last 6 weeks, mice were injected intraperitoneal with 100 μg of anti-ICOSL (clone HK5.3) or isotype control (Rat IgG2a) (BioXcell, West Lebanon, NH) twice a week, similar to Choi et al.^30^. Mice were sacrificed and Tfh populations were assessed in aorta, peri-aortic LNs and spleen as described earlier.

### *Bcl6* deficient *LDLR*^*−/−*^ chimera generation

Femurs from B6, (WT; control) and *Bcl6*^*fl/fl-*^*CD4Cre*^+^ mice were centrifuged to collect the bone marrow cells in PBS. Recipient *LDLR*^*−/−*^ mice were irradiated with 550 rads twice, 4 h apart. Approximately 1 × 10^7^ bone marrow cells were injected retro-orbitally into recipient mice. Mice were allowed to reconstitute for 6 weeks before they were fed a high-cholesterol diet for 15 weeks.

### Atherosclerosis quantification

Aortic roots were dissected and snap frozen in OCT over dry ice. Serial frozen sections were cut at 10 μm thickness and stained with Oil Red O (for neutral lipids) and in some cases counterstained with hematoxylin (for nuclei) using standard procedure. At least four sections per aortic root per mouse were stained and scanned with Zeiss AxioScan Z1 slide scanner at 20 × magnification. The area of red pixels was determined by Image-Pro Analyzer software 7.0.1 and the pixel area values were converted to μm^2^. For en face staining, mouse aortae were collected and immersed in paraformaldehyde and stained with Oil Red O, open longitudinally and pinned as previously^65^. Images were scanned and the percentage of lesion surface area was determined with Photoshop software.

### Immunofluorescence staining of CD68^+^ cell in aortic roots

Aortic roots described above where stained for macrophages using immunofluorescence using purified rat anti-CD68 (clone FA-11, 5 μg ml^−1^, Biolegend), following by goat anti-rat-AF568 (4 μg ml^−1^, ThermoFisher) and counterstained with phalloidin AF647 for actin (ThermoFisher) and Hoechst^®^ for nuclei (ThermoFisher). Isotype control sections were used to set thresholds. Images were acquired using Zeiss AxioScan Z1 slide scanner and analyze with Image J software. 4 sections were used per mouse and the average area of all four sections was used to calculate the CD68^+^ area.

### Plasma cholesterol and HDL levels

The lipoprotein cholesterol distribution was determined in the plasma of LT-ApoE^*−/−*^ mice following fasting for 4 h by liquid chromatography (FPLC) as previously described^23^.

### Mass spectroscopy for intracellular lipids

To determine the cholesterol content in the cells, current Treg (RFP^+^YFP^+^) from LT-ApoE^*−/−*^ chow or western diet fed, with and without administration of ApoAI, were sorted from peri-aortic LNs, washed and cell pellets were frozen at −80 °C till analyzed. Cholesterol was extracted from the cells and the lipids extracts were analyzed by mass spectroscopy as previously described^70^.

### Quantitative real time PCR

Total cellular RNA from sorted current or exTreg from LT-ApoE^*−/−*^ mice was extracted by Trizol (Life Technologies) followed by RNA purification using Direct-zol^TM^ RNA miniPrep (Zymo Research, Irvine, CA) per manufacturer’s instructions. RNA purity and quantity was determined by nanodrop spectrophotometer (Thermo Scientific) and equal amounts of RNA was used to synthesis cDNA using iScript cDNA synthesis kit (Bio-Rad). mRNA expression was measured in real time quantitative PCR using TaqMan Gene Expression system and predesigned TaqMan primers for *Foxp3*, *Blimp1*, *Bcl6*, *Il21*, *IFNγ, ABCA1*, and *β-actin* (Applied Biosytems). Data were analyzed and presented on the basis of relative expression method. The 2^-^^ΔΔCT^ method was used with *β-actin* as a housekeeping gene.

### ELISA

Plasma samples were collected from LT-ApoE^*−/−*^ mice using cardiac puncture and EDTA coated syringes. Samples were centrifuged to remove RBCs and frozen at −80 °C till analyzed. Supernatants from in vitro Treg induction were collected following 36 h of stimulation and frozen at −80 °C till analyzed. Both mouse IL-6 and IL-2 ELISAs were performed using Ready-Set-Go ELISA kits (ebioscience) according to manufacturer’s instruction.

### Human samples

PBMCs and plasma sample were isolated from blood of donors at the Cardiac Catheterization laboratory in University of Virginia, Charlottesville. Enrolled participants included males and females of different ages (for human subjects characteristics, see Supplementary Table 1) and informed consent was obtained from all subjects. Studies were performed with the approval of the Institutional Review Board of Health Science Research at the University of Virginia. Blood was drawn into vacutainer EDTA (BD Bioscience), spun and the top plasma layer was collected and frozen at −80 °C until analyzed. Thawed plasma samples were tested for the levels of ApoAI and IL-21 using human ApoAI (ABCAM, Cambridge, MA) and IL-21 ELISA (ebioscience) kits, respectively, according to manufacturer’s instruction. Peripheral blood mononuclear cells (PBMC) were isolated from blood that was depleted from plasma with Ficoll-Paque™PLUS (GE Healthcare) and SepMate™ tubes (STEMCELL Technologies, Inc.). For Treg percentages, Treg were identified as CD4^+^CD25^+^CD127^−^ [and/or Foxp3^+^] cells by flow cytometry. Antibodies used were anti-CD4 (clone OKT4; 1 test per sample, Tonbo Bioscience), anti-CD25 (clone BC96; 1 test per sample, Tonbo Bioscience), anti-CD127 (clone A019D5, 1 test per sample, Biolegend), and anti-Foxp3 (clone PCH101, 0.5 μg per test ebioscience).

### Statistical analyses

All results are expressed as mean ± s.e.m. Results were analyzed by either unpaired two-tailed Student’s *t*-test, one-way analysis of variance (one way ANOVA), or two-way ANOVA, followed by Tukey’s multiple comparisons test. Our sample size was based on previous studies, consulting with biostatisticians, and using nQuery Advisor 6.0 software. Via this analysis, we found that 90% power with 1% type 1 error can be achieved with 8 mice per group for atherosclerosis quantification. For in vitro and qRT-PCR studies, no statistical method was used to determine sample size. No exclusion was performed. The data seemed to be normally distributed with similar s.d. and error observations between experimental groups and controls. A *P* value of <0.05 is considered to be significant. Statistical analysis was performed using GraphPad Prism software version 6 (GraphPad Software, Inc.). Linear regression and Pearson correlation coefficient (*r*) were used to determine the statistical significance and correlation in the human studies.

### Data availability

The datasets generated during and/or analyzed during the current study are available from the corresponding author upon reasonable request.

## Electronic supplementary material

Supplementary Information
