# Supplementary material for: Apolipoprotein AI prevents regulatory to follicular helper T cell switching during atherosclerosis
Source: Nat Commun. 2018 Mar 15;9:1095. doi: 10.1038/s41467-018-03493-5 (PMC5854619; doi:10.1038/s41467-018-03493-5)
Supplement: Supplementary file 1 — Supplementary Information [file 41467_2018_3493_MOESM1_ESM.pdf]

**Supplementary Information**

# Apolipoprotein AI prevents regulatory to follicular helper T cell switching during atherosclerosis

**Gaddis et al.**

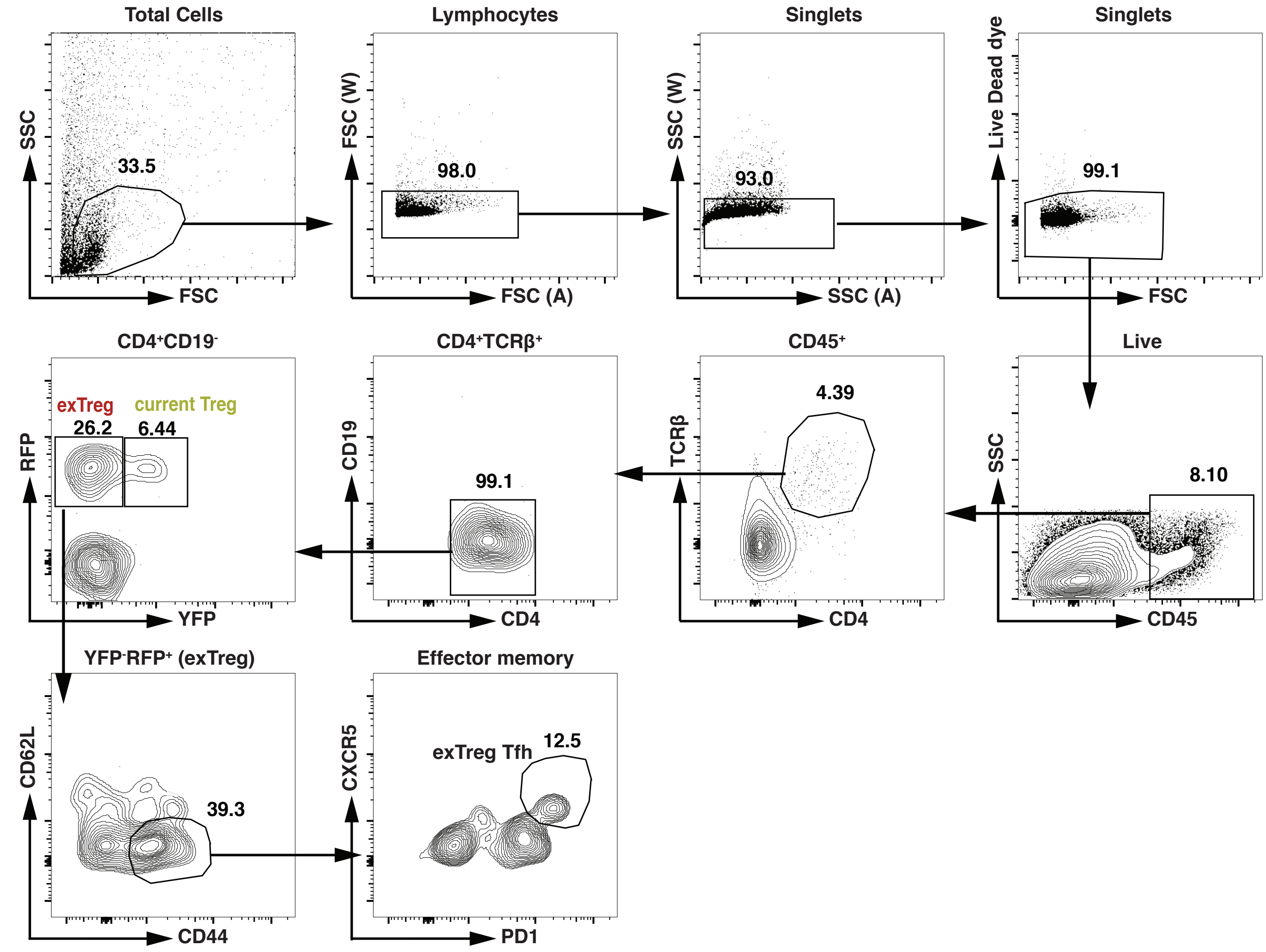


**Supplementary Figure 1: Gating Strategy of current Treg, exTreg and Tfh from exTreg cells in the aorta of western diet-fed LT-ApoE$^{-/-}$ mice.** Flow cytometry plots showing gating strategy for current Treg, exTreg and Tfh from exTreg cells in the aorta of WD-fed LT-ApoE-/- mice. Similar gating strategy was performed for PaLN and spleens excluding the CD45 staining. Results show a representative mouse.

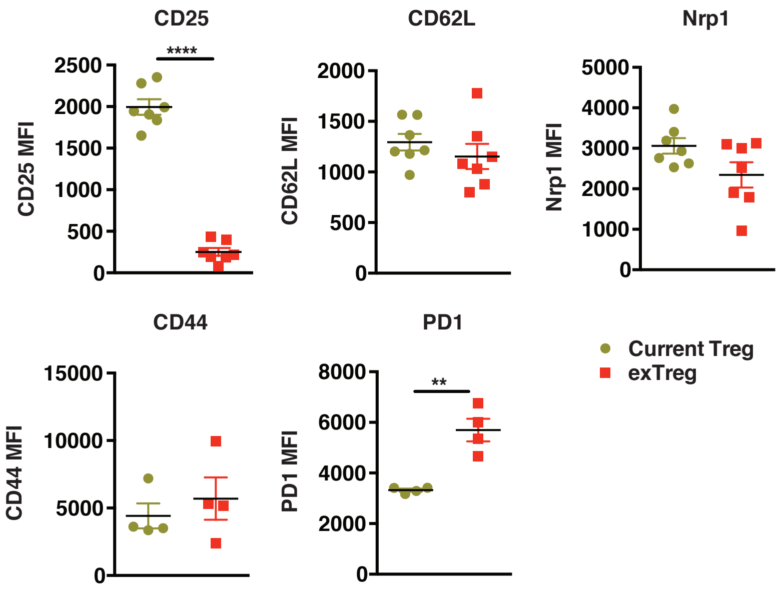


**Supplementary Figure 2: Expression of surface markers on current and exTreg cells.** Current and exTreg cells from western diet-fed LT-ApoE$^{-/-}$ mice were evaluated for the expression of CD25, CD62L, Nrp1, CD44 and PD1. Graphs show the mean fluorescence intensity of each molecule on both cell subsets. Results are expressed as the mean ± s.e.m from one experiment (n=4-7). Statistical significant differences were at * $P < 0.05$, ** $P < 0.01$ and **** $P < 0.001$ (Unpaired Student's *t*-test).

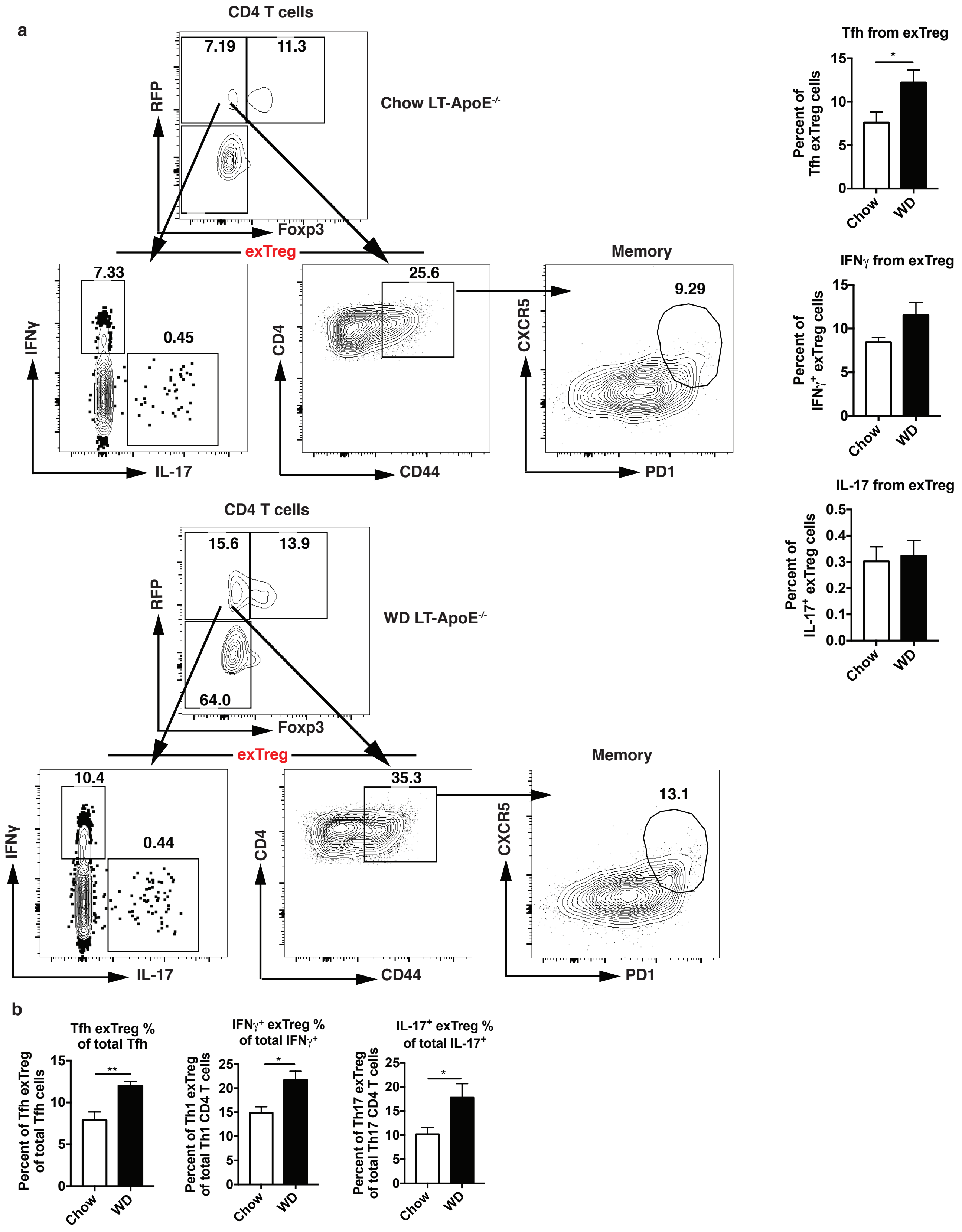


**Supplementary Figure 3: Distribution of the different T cell subsets within the exTreg population.** PaLN from LT-ApoE$^{-/-}$ mice on western diet and chow controls were examined for the distribution of the different T cell subsets within the exTreg population. (**a**) Flow plots and graphs show the percentages of memory, Th-1, Th-17 and Tfh cells within the exTreg population. (**b**) Graphs showing the percentages of each exTreg population within the total population of cells. Results are expressed as mean ± s.e.m. from one experiment (n=7 (chow) and n=8 (WD)). Statistical significant differences were at * $P <$ 0.05, and ** $P < 0.01$ (unpaired Student's *t*-test).

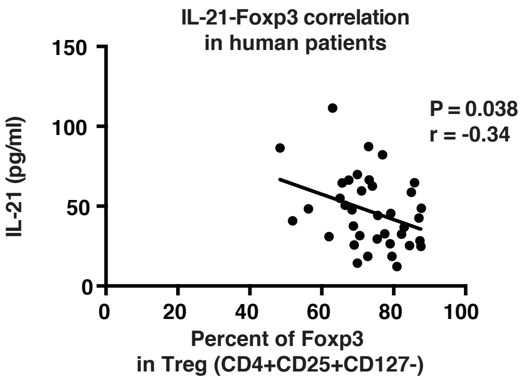


**Supplementary Figure 4: In CAD patients, IL-21 levels inversely correlates with Foxp3 expression.** Graph shows a negative correlation between plasma levels of IL-21 and Foxp3 expression on Treg from PBMCs of human patients. Correlation was determined using linear regression and the results were statistically significant at $P = 0.038$.

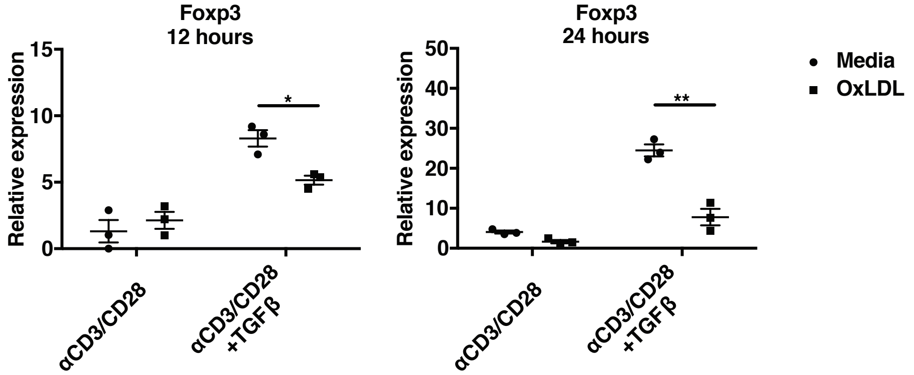


**Supplementary Figure 5: OxLDL reduces *Foxp3* mRNA expression.** Naïve CD4 T cells from ApoE$^{-/-}$ mice were isolated and stimulated with αCD3/CD28 and TGFβ to induce Treg *in vitro* with or without the addition of oxLDL. RNA was harvested at 12 and 24 hours post-stimulation and the levels of *Foxp3* mRNA were detected by qRT-PCR. Results are expressed as the mean ± s.e.m. from one experiment (n=3). Statistical significant differences were at * $P < 0.05$ and ** $P < 0.01$ (Unpaired Student's *t* test).

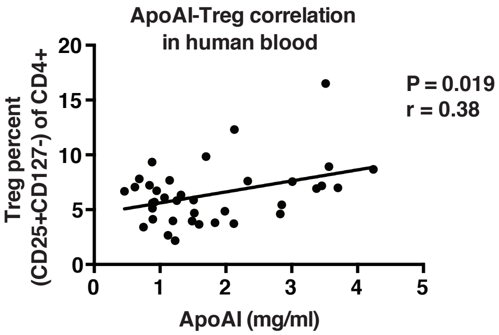


**Supplementary Figure 6: In CAD patients, ApoAI levels positively correlates with Treg percentages in blood.** PBMC and plasma samples from human patients were assessed for the percentages of Treg and levels of plasma ApoAI. A positive correlation of the percentages of Treg cells and ApoAI plasma levels was determined using linear regression and the results were statistically significant at $P = 0.019$.

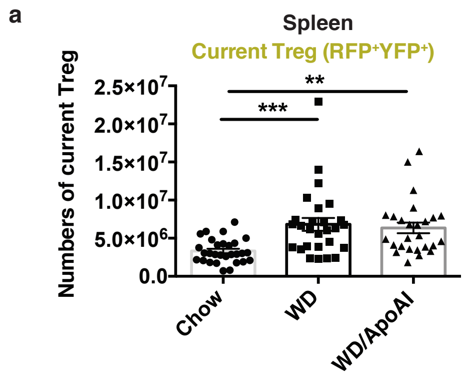


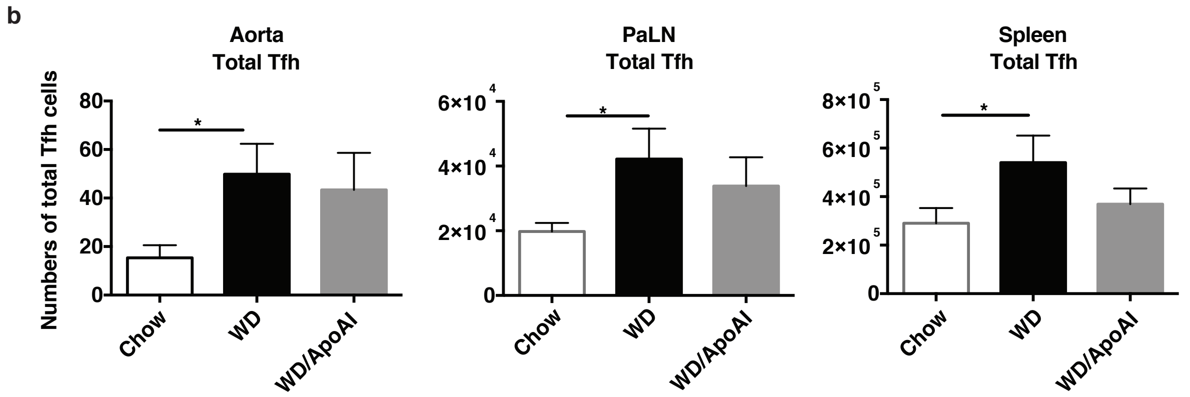


**Supplementary Figure 7: Treg and total Tfh cells number in ApoAI treated LT-ApoE$^{-/-}$ mice.** Numbers of Treg cells in the spleen **(a)** and total Tfh cells in the aorta, PaLN and spleen **(b)** of LT-ApoE$^{-/-}$ western diet-fed mice with and without ApoAI treatment or chow controls. Results are expressed as the mean ± s.e.m. from four independent experiments (n=29 (Chow), n=27 (WD) and n=26 (WD/ApoAI) **(a)**, and from three independent experiments (n=19 (Chow), n=16 (WD & WD/ApoAI)) **(b)**. Statistical significant differences were at * $P < 0.05$, ** $P < 0.01$, *** $P < 0.001$ (one-way Anova).

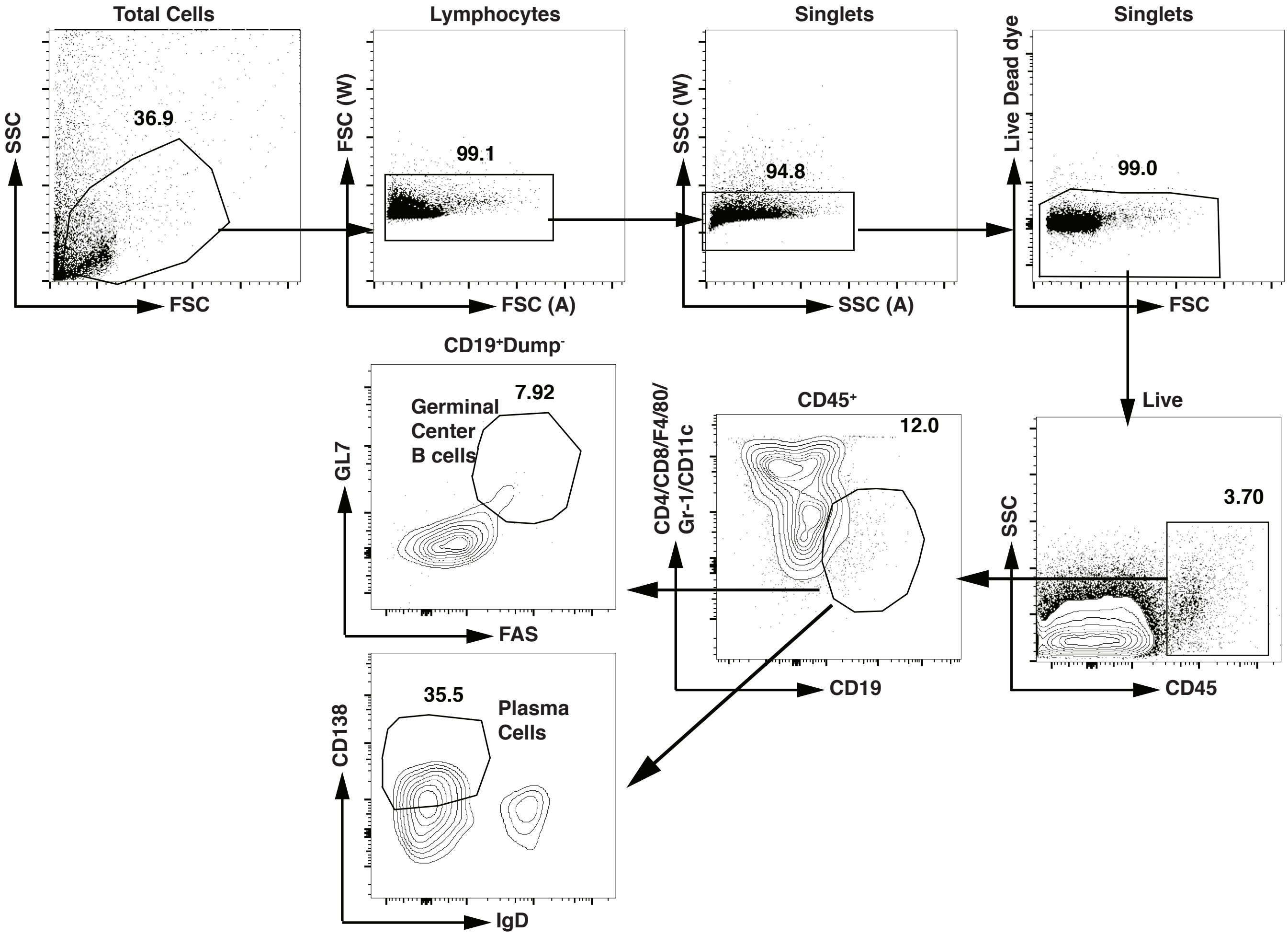


**Supplementary Figure 8: Gating Strategy of germinal center B cells and plasma cells in aorta.** Flow cytometry plots showing gating strategy for germinal center B cells and plasma cells in the aorta of WD-fed LT-ApoE-/- mice. Similar gating strategy was performed for PaLN and spleens excluding the CD45 staining. Results show a representative mouse.

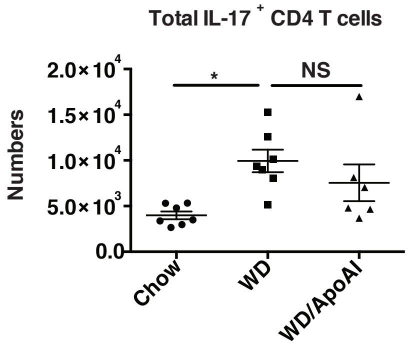


**Supplementary Figure 9: ApoAI administration does not reduce total IL-17⁺ CD4 T cells in the PaLN.** Numbers of IL-17⁺ from total CD4 T cells in the PaLN of LT-ApoE⁻/⁻ western diet-fed mice with and without ApoAI treatment or chow controls, following stimulation with PMA/ionomycin for 5 hrs. Results are expressed as the mean ± s.e.m from one experiment (n=6-7). Statistical significant differences were at * $P < 0.05$ (one-way Anova).

**Supplementary Table 1. Human Subjects Characteristics.**

| Patient Characteristics | Cohort | % |
|---|---|---|
| **Number of subjects** | 37 | |
| | | |
| **Women** | 18 | 48.6% |
| **Men** | 19 | 51.4% |
| | | |
| **White** | 37 | 100.0% |
| **African-American** | 0 | 0.0% |
| **Hispanic** | 0 | 0.0% |
| **Non-Hispanic** | 37 | 100.0% |
| | | |
| **Median Age** | | |
| Women (years) | 62.0 [49 - 75] | |
| Men (years) | 62.0 [40 - 78] | |
| | | |
| **Median BMI (kg/m)** | 28.4 [17.2 - 50.0] | |
| **Lipid Medication, n (%)** | 30 | 81.0% |
| **LDL-Cholesterol, mg/dL** | 92.0 [59 - 166] | |
| **HDL-Cholesterol, mg/dL** | 41.0 [22 - 71] | |
| **Triglycerides, mg/dL** | 80 [40 - 283] | |
| **Maximum Stenosis in Coronary Artery (%)** | 85% [0% - 100%] | |
